# Nest survival patterns in Eurasian Bittern: effect of nest age, time and habitat variables

**DOI:** 10.7717/peerj.2047

**Published:** 2016-06-16

**Authors:** Marcin Polak

**Affiliations:** Department of Nature Conservation, Institute of Biology and Biochemistry, Maria Curie-Sklodowska University Lublin, Lublin, Poland

**Keywords:** Nest survival predation waterbirds

## Abstract

Determining the key factors affecting the reproductive success of nesting birds is crucial in order to better understand the population dynamics of endangered species and to introduce effective conservation programmes for them. Inhabiting a variety of wetland habitats, aquatic birds actively select safe nesting sites so as to protect their nests against predators. The main aim of the present work was to assess the effect of temporal and habitat variables on the daily nest survival rate of Eurasian Bitterns colonizing semi–natural fishpond habitat in eastern Poland. MARK software was used for the modelling. Eurasian Bittern nests were most vulnerable to depredation at the beginning of the breeding season. This was probably because the reedbed vegetation at this time was not yet dense enough to effectively conceal the nests. There was a positive relationship between nest age and the daily survival rate. Two of the habitat variables analysed were of the greatest significance: water depth and vegetation density. In the Eurasian Bittern population studied here, nests built over deep water and in dense vegetation had the best chances of survival. The results of this work may be useful in the preparation of plans for the conservation and management of populations of this rare and endangered species. Conservation and restoration efforts that attempt to maintain high water levels will be especially beneficial to this avian species that is dependent on wetland ecosystems for breeding.

## Introduction

Nest predation is the basic reason for the lack of reproductive success in most bird species (Martin, 1992). This factor is thought to be responsible for an average of 80% of all nest losses (Martin, 1993). Reproductive success in birds depends to a large extent on the intensity of predator activity and pressure on the one hand, and on the abilities of both parent birds and nestlings to cope with this threat on the other (Caro, 2005; Ibáñez-Álamo et al., 2015). Birds apply a wide variety of anti-predator adaptations and strategies (Lima, 2009). In order to prevent their clutches from being plundered, adult birds have to make the key decisions where and exactly when in the annual cycle to breed (Hoover, 2006; Lewis et al., 2012). Studies to date have tended to concentrate on assessing the influence of habitat quality and the differentiation in vegetation structure on the level of nest predation (Sanchez-Lafuente, Alcántra & Romero, 1998; Goławski & Mitrus, 2008); rather less attention has been paid to the temporal aspect of brood survival (Wilson, Martin & Hannon, 2007). In recent years, an extensive suite of modern analytical tools has become available that enable the effect of season and nest age, in combination with other habitat variables, on the nesting success of birds to be modelled more precisely (White & Burnham, 1999; Dinsmore, White & Knopf, 2002; Rotella, Dinsmore & Shaffer, 2004). The present study explores these problems with respect to one of the European heron species, the Eurasian Bittern (henceforth Bittern) *Botaurus stellaris*. This species is especially suitable for examining the issues mentioned above for the following reasons: (1) Bitterns have a very prolonged breeding season (Mallord et al., 2000; Demongin, Dmitrenok & Bretagnolle, 2007); (2) they nest in a wide spectrum of wetland habitats with varied vegetation structures and hydrological regimes (Tyler, Smith & Burges, 1998; Polak, Kasprzykowski & Kucharczyk, 2008); (3) recent studies have indicated that the Bittern is a flexible species, readily adapting to local environmental conditions and variable predation pressure (Adamo, Puglisi & Baldaccini, 2004; Gilbert et al., 2005; Kasprzykowski, Polak & Chylarecki, 2014); (4) in natural and semi-natural habitats the major cause of brood losses in Bitterns is nest predation (Polak & Kasprzykowski, 2010).

The principal objective of the present study was to describe habitat variables at nests and define the factors significantly affecting the daily nest survival of Bitterns colonizing a fishpond environment. Fishpond complexes are an important habitat for this species in Central and Eastern Europe (White, Purps & Alsbury, 2006; Polak & Kasprzykowski, 2010). Such a study is of major importance, given that in some European countries the Bittern is an endangered species whose numbers are declining as a result of habitat degradation (White, Purps & Alsbury, 2006). This study attempts to test the effect of following temporal and habitat variables:

(1) *Intra-season effect*. In most studied bird populations, the risk of nest predation is not constant across the breeding season (Wilson, Martin & Hannon, 2007). Some authors have noticed that because of the ever more vigorous defence of broods on the part of parent birds, daily nest survival rate increases as the season progresses (Segura & Reboreda, 2012). Other studies have demonstrated a reverse linear trend, where the level of predation is least at the start of the season, after which it gradually rises (Elmberg et al., 2009). Again, in some species it has been observed that daily survival can vary as a quadratic function and that the risk of predation may be greatest or least in the middle of the breeding season (Smith & Wilson, 2010; Lewis et al., 2012). In the case of aquatic birds inhabiting reedbeds, one can assume that the taller and denser the reedbeds, the better the nests are concealed (Jedlikowski, Brzeziński & Chibowski, 2015); hence, due to seasonal vegetation development daily nest survival ought to increase as the breeding period progresses.

(2) *Nest age*. Most studies indicate that the older the nest, the higher the daily survival level (Dinsmore, White & Knopf, 2002). The assumption underlying the present study was that predators would quickly discover badly concealed nests in the early stages of reproduction, whereas well-hidden nests would survive longer (Martin, Scott & Menge, 2000).

(3) *Water depth*. The extent to which the nests of birds breeding in wetlands are depredated falls with increasing water depth at the nesting site (Sanchez-Lafuente, Alcántra & Romero, 1998). Deep water can set up an effective barrier, especially to mammalian predators depredating the broods of wetland and marshland birds (Hoover, 2006). It was therefore to be expected that Bittern nests built over deep water should be more successful than nests built where the water is shallower.

(4) *Height and density of vegetation*. A significant factor enhancing reproductive success in birds colonizing reedbed habitats is the dense vegetation that makes for better nest concealment (Kristiansen, 1998). In the case of terrestrial (mainly mammalian) predators, the density of the plant cover should be more important, whereas in the case of flying predators (mainly raptors and corvids) the height of the reedbeds is likely to be more important (Jedlikowski, Brzeziński & Chibowski, 2015). This study tested the prediction that nests in tall, dense vegetation would have greater chances of survival than nests in shorter, sparser vegetation.

(5) *Fragmentation of habitats (the size of vegetation patches)*. Recent studies have demonstrated a positive relationship between the level of nest predation and habitat fragmentation (Caro, 2005). Mosaic-like habitats and the well-developed ecotone zones within them may be attractive to predators and facilitate their search for birds’ nests. One can therefore expect that nests situated in smaller patches of vegetation ought to be in greater danger of predation than nests in more extensive reedbeds.

## Materials & Methods

### Field data collection

I carried out the fieldwork in ten fishpond complexes in the Lublin region in eastern Poland. The fishpond complexes varied in size from 15 to 185 ha and were partially covered by vegetation stands dominated by Common Reed *Phragmites australis*, Reed Mace *Typha angustifolia* and sedges *Carex* sp. The water depth in the emergent vegetation varied from 0 to 120 cm. The ponds were similar in depth (from 0.7 to 1.3 m), but they differed strongly in surface area and coverage of emergent aquatic vegetation. They were filled with water from adjoining rivers and streams or by precipitation. The ponds were surrounded by arable fields, meadows and small villages and woodland areas of different ages.

I collected data on the timing of breeding, brood size and breeding success of Bitterns in 2003–2008, surveying the study fishponds from the pre-breeding period (the beginning of March) to early July at least once a week. I plotted the movements of the birds, locations of booming males and active nests on 1:5000 maps and located nests by walking along transects within the emergent vegetation. I paid special attention to searching for nests within or near booming areas of males, because in the studied population most of nesting females located nests in the male’s territories (M Polak, 2003–2008, unpublished data). I subsequently visited all 103 active nests at least once a week from the end of April to early July in order to obtain data on breeding characteristics (mean number of inspections = 4, range = 1–9). To reduce the impact of nest visits on predation risk, I kept the number of inspections to a minimum. I ringed nestlings for individual recognition with metal and coloured rings, and I defined the clutch initiation date as the day when the first egg was laid. I was able to determine laying dates by direct observation (ca. 30% of nests), or indirectly, by estimating the hatching date of the oldest nestling, assuming an incubation period of 25–26 days (Mallord et al., 2000; Demongin, Dmitrenok & Bretagnolle, 2007). I defined nests as successful if at least one young bird survived up to 15 days old. I established the number of nestlings during the first inspection of a nest after hatching.

I measured habitat variables using a modified version of the methodology applied in studies of the Bittern in the UK (Tyler, Smith & Burges, 1998; Gilbert et al., 2005). I examined eight different temporal and habitat variables (Table 1). Providing a very good description of the Bittern’s habitat use, these variables may determine the reproductive success of this species in specific semi-natural habitats like fishponds (Polak, Kasprzykowski & Kucharczyk, 2008). In particular; the ratio of open water area to emergent vegetation area is a much better habitat descriptor of a fishpond than other macrohabitat components like the length of water edge, which are important for populations living in more natural habitats. I made all the measurements between the end of April and the end of May: I measured the microvariables in within 50 x 50 cm squares placed around the nests and the macrovariables, including the areas of the reed patches in which the nests occurred, from aerial photographs (GEOPORTAL, www.geoportal.gov.pl). For a detailed description of the study area and methods, see Polak, Kasprzykowski & Kucharczyk (2008), Polak & Kasprzykowski (2010) and Polak & Kasprzykowski (2013).

**Table 1:** Description of variables used to model nest survival of Eurasian Bitterns on fishponds in eastern Poland, in 2003–2008.

Code	Meaning
YEAR	Code of year
SITE	Code of fishpond complex
WATER	Estimated water depth (cm) at the centre of the plot with 1–cm precision
DENSITY	Number of stems within a 50 × 50 cm square
HEIGHT	Mean height of 5 stems chosen randomly with 10–cm precision within a 50 × 50 cm square
DISTOW	Distance (m) to open water
DISTDYKE	Distance (m) to the fishpond dyke
PROARE	Ratio of open water area (ha) to emergent vegetation area (ha) on a given fishpond
TIME	Date of breeding (days)
AGE	Nest age (days)

### Modelling

I used the nest survival module in version 8.0 of the MARK program (White & Burnham, 1999; Rotella, Dinsmore & Shaffer, 2004) to compare nest survival models and to obtain estimates of daily nest survival. All the analyses performed in MARK included broods with success and nests lost only as a result of predation. I scaled the dates such that 1 was the date when I found the first nest. I thus defined a 79-day nesting season beginning on 19 April and ending on 6 July. Consequently, the season consisted of 78 daily intervals for which I estimated the daily survival rate. I selected the best predictive models using Akaike’s information criterion corrected for a small sample size (AIC_*c*_; Burnham & Anderson, 2002). The list of candidate models was based on combinations of factors that I assumed *a priori* might affect Bittern nest survival. I compared the model support using AIC_*c*_ and evaluated the strength of evidence for each model using normalized weights w_*i*_. I followed the convention that the model with the lowest AIC_*c*_ represented the best compromise between goodness-of-fit and model complexity (Whittingham et al., 2006). Because the candidate model set contained a mix of models with linear and quadratic terms, as well as interactions, I was unable to use model averaging in the interpretation of variable estimates (Wilson, Martin & Hannon, 2007). Firstly, I considered the influence of habitat variables. I constructed models of nest survival that incorporated combinations of individual variables and compared them to the null model of constant survival rate S (.). I expected that the better concealed nests (denser vegetation) would have a greater chance of survival than the more exposed ones (Goławski & Mitrus, 2008). The decision when to initiate breeding is of crucial significance where the adaptation of individuals is concerned (Lewis et al., 2012). Due to severe winter weather, in eastern Europe Bitterns usually have only a short time window for start of breeding, and these individuals are under strong pressure from the passage of time (Polak & Kasprzykowski, 2010). The daily survival rate of many avian species varies in relation to nest age and may decline as a result of the greater frequency of visits by parents when feeding nestlings and increase as a result of intensive nest defence (Martin, Scott & Menge, 2000). Recent studies have indicated, however, that during the nestling period female Bitterns do not defend their broods or behave aggressively towards predators (White, Purps & Alsbury, 2006). Nest age and day of season can be difficult to separate when nests are initiated synchronously, but as most nests were initiated across the season, I included both variables in the models. As suggested by Dinsmore, White & Knopf (2002), all were unstandardized, because the unstandardized variables did not affect numerical optimization. Since a goodness-of-fit test is not yet available in MARK for nest survival models, I did not adjust for overdispersion. The logit link function was adopted. Unless otherwise indicated, means are expressed ±SD and all tests are two-tailed. The study fulfilled the current Polish Law and was permitted by the 1st Local Ethic Commission in Lublin (approval number: 514/2005). The study was permitted by Ministry of the Environment (approval number: DOPog–4201–03–48/05/al.). Provincial Nature Conservator in Lublin allowed for this research project by the letter (approval number: ŚR.IV.6631–ZW/70/2003).

## Results

I monitored a total of 103 Bittern nests in this study. Of these, however, 63 successful nests and 31 depredated by predators were chosen for analysis with the MARK program. The mean daily survival rate calculated for these 94 nests was 0.986 ± 0.003 SE (95% CI [0.98–0.99]). Eight (26%) of the depredated nests were destroyed during the egg-laying stage, 17 (55%) during incubation and 6 (19%) during the nestling period. All the nest sites were surrounded by water (mean = 44.6 ± 18.0 cm; range 10–97 cm; *n* = 94). The height of the reedbed vegetation at the nesting sites varied from 1.7 to 3.4 m (mean = 2.4 ± 0.4 m; *n* = 94), and the mean density of the vegetation was 31.5 ± 21.4 (range 0–85; *n* = 94). Nests were located on average 22.5 ± 14.0 m from open water (range 4–70 m; *n* = 94) and 21.6 ± 18.4 m from the dyke (range 6–100 m; *n* = 94). Female Bitterns built their nests on fishponds with a varied habitat structure and variable areas of reedbed patches (mean = 2.8 ± 2.1 ha; range 0.1–10 ha; *n* = 94). The ratio of open water area to reedbed area in the different ponds varied widely from 0.1 to 19.0 (mean = 2.1 ± 2.7; *n* = 94).

**Figure 1:**
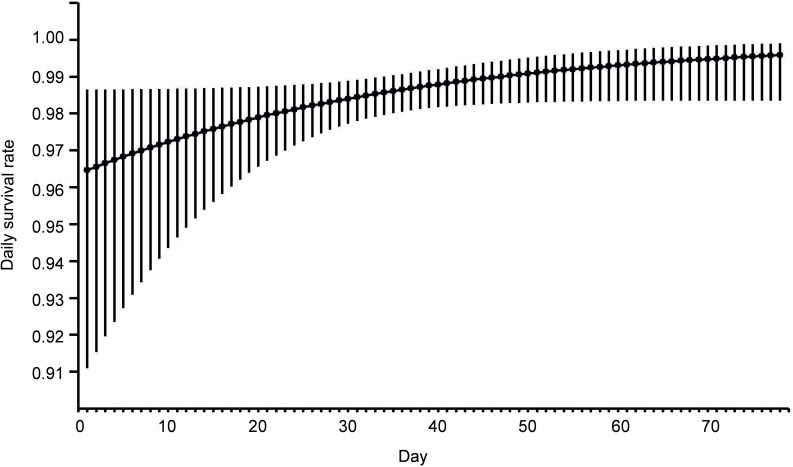
Model averaged estimates of daily nest survival in eastern Poland, 2003–2008, for Eurasian Bittern showing effect of day in the breeding season. Solid line represents daily survival rate estimated using beta parameters from the best–fit model. Vertical lines represent upper and lower 95% confidence intervals for the estimated daily survival rate.

**Figure 2:**
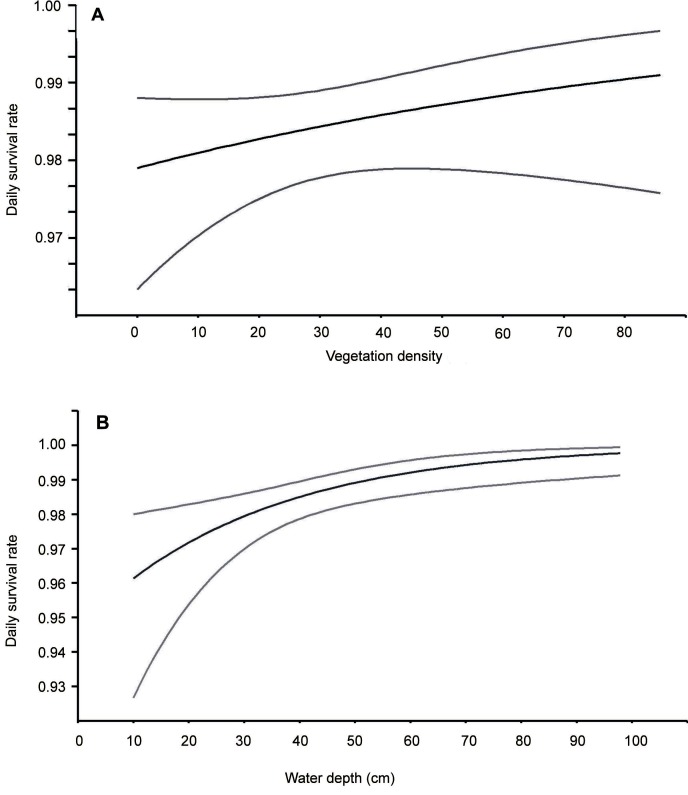
Daily nest survival rate for Eurasian Bittern in relation to: (A) vegetation density in the vicinity of nest, (B) water depth at nest. Middle lines represent daily survival rate. Upper and lower lines represent upper and lower 95% confidence intervals for the estimated daily survival rate.

This analysis showed that both temporal and habitat variables affected the risk of these nests being depredated (Table 2). Models with the highest ranking predicted that the survival of Bittern broods gradually increased with nest age and date of breeding season. Nests initiated at the beginning of the breeding season had the smallest chances of survival (Fig. 1). The best models showing a linear trend were more strongly supported than those displaying a quadratic effect. The habitat factors with the greatest influence on the likelihood of nest depredation were vegetation density and the depth of water around the nest. The models with these two variables achieved the lowest AIC values in the ranking. Nests built over deeper water and better concealed by the vegetation were less exposed to predation (Fig. 2). The analysis showed that macrohabitat factors had no effect on the survival of Bittern broods. There was greater support for the null model of constant survival rate S (.) in the set of models assessing the effect of year and site, which indicates that the daily survival rate of Bittern nests did not vary between the particular fishpond complexes and years.

**Table 2:** Top candidate models predicting nest survival of Eurasian Bitterns in eastern Poland. The number of variables (*k*), Akaike’s information criterion with small-sample bias adjustment (AIC_*c*_), the difference between the lowest AIC_*c*_ and AIC_*ci*_($\mrm{\Delta }$AIC_*c*_), and the model weight (w_*i*_) are shown.

Candidate model	*k*	AIC_*c*_	$\mrm{\Delta }$AIC_*c*_	w_*i*_
Eurasian Bittern (*n* = 94 nests)				
S (TIME + AGE + DENSITY + WATER)	5	159.85	0.00	0.231
S (TIME + AGE )	3	162.42	2.57	0.064
S (TIME + AGE + AGE^2^)	4	163.38	3.53	0.040
S (TIME + AGE + WATER)	4	163.67	3.81	0.034
S (AGE + WATER + DENSITY)	3	164.28	4.43	0.025
S (AGE^2^ + WATER + DENSITY)	3	164.70	4.85	0.020
S (TIME + TIME^2^ + AGE + AGE^2^)	5	164.93	5.08	0.018
S (TIME^2^ + AGE + AGE^2^)	4	165.17	5.32	0.016
S (WATER + DENSITY)	3	165.28	5.43	0.015
S (AGE)	2	166.53	6.68	0.008
S (WATER)	2	166.58	6.73	0.008
S (AGE + AGE^2^)	3	168.32	8.47	0.003
S (TIME + DENSITY + WATER)	3	169.62	9.77	0.002
S (TIME)	2	172.72	12.87	0.000
S (TIME^2^)	2	173.53	13.68	0.000
S (TIME + TIME^2^)	2	174.21	14.36	0.000
S (.)	1	174.39	14.54	0.000

## Discussion

### Effect of season

Numerous studies have shown that early nesting during the breeding period is advantageous to many species of birds (Wesołowski, 1998), including herons (Jakubas, 2011), ducks (Elmberg et al., 2009) and other aquatic birds (Lewis et al., 2012). On arrival from their wintering grounds, early breeders are the first to occupy the best breeding territories, which offer abundant food resources and safe breeding sites (Forsman et al., 1998). A gradual decline in the values of reproductive characteristics (clutch size, egg volume, breeding success) during the breeding season has been demonstrated in many bird species (Borgmann, Conway & Morrison, 2013), including herons (Moser, 1986; Jakubas, 2011). This may be due to the fact that after their return from the wintering grounds, the first to begin nesting are birds in good genetic and phenotypic condition, which outcompete younger and less experienced individuals (Lewis et al., 2012). The quickest possible start to breeding is therefore of the utmost importance, especially in species with prolonged parental care (Tobółka, Żołnierowicz & Reeve, 2015). On the other hand, some studies have shown that early nesting can have negative consequences for individuals choosing this strategy (Janiszewski, Minias & Wojciechowski, 2013). Previous work (Polak & Kasprzykowski, 2013; Kasprzykowski, Polak & Chylarecki, 2014) indicates that an early start to breeding by female Bitterns is not a successful strategy in this study area. In the temperate climate zone the beginning of the season is often a time of inclement weather with below-zero temperatures, strong winds and precipitation, which can significantly affect the reproductive characteristics of nesting birds (Jakubas, 2011; Tobółka, Żołnierowicz & Reeve, 2015). In the Bittern population I studied here, intensive rainfall during the pre-breeding period adversely affected the size of eggs laid by the females (Polak & Kasprzykowski, 2013). Previous studies had also shown that nestlings from later nests gained weight more quickly in comparison with young birds from early nests (Kasprzykowski, Polak & Chylarecki, 2014). The present study showed that the risk of nest predation in this population was greatest at the beginning of the season and that clutches laid early were more vulnerable to depredation. On their return from the wintering grounds female Bitterns have difficulty in finding suitable sites for concealing their nests, because in the reedbeds around the fishponds reed and bulrush shoots have yet to start growing; they are therefore forced to choose nesting sites in the previous year’s vegetation. This accords with earlier studies, which showed that female Bitterns preferred reedbeds with a high density of last year’s stems within which to conceal their nests (Polak, Kasprzykowski & Kucharczyk, 2008).

### Nest age

Most work done to date has shown that the older the nest, the greater the chances of the brood fledging (Dinsmore, White & Knopf, 2002; Segura & Reboreda, 2012). The degree of aggression on the part of the parents towards potential predators usually varies during the breeding season (Caro, 2005). The intensity with which they defend their nest grows as the investment they make in their brood increases (Lima, 2009). Moreover, the probability of fledging of young birds from replacement clutches gradually decreases as the season progresses (Janiszewski, Minias & Wojciechowski, 2013). On the other hand, some researchers have observed the opposite trend, namely, that the predation level increases during the chick-feeding period, since the adults’ frequent foraging flights and feeding their chicks heightens the risk of their brood being discovered by predators (Martin, Scott & Menge, 2000). Distinguishing the nest age factor from the effect of season, which simultaneously alters the level of nest survival, is particularly difficult in the case of birds that have a short, synchronized pattern of breeding onset phenology (Dinsmore, White & Knopf, 2002). Earlier observations showed, however, that in this study area Bitterns have quite a prolonged reproductive period (Polak & Kasprzykowski, 2010) and this enables the influence of these two factors to be better differentiated in the final analysis. Unlike other more aggressive species, female Bitterns do not exhibit intensive anti-predator behaviour near the nest (White, Purps & Alsbury, 2006). They are cryptically coloured and sit motionless on the nest when danger threatens, in this way reducing their chance of being detected by a predator. In this case intensive brood defence in the nestling period was not a significant factor enhancing brood survival in Bitterns in the later stages of their reproduction. It is possible that on these fishponds the most conspicuous nests were quickly discovered by predators during the egg-laying and incubation periods, while well-concealed nests were less subject to nest predation (Martin, Scott & Menge, 2000; Dinsmore, White & Knopf, 2002).

### Water depth

Many studies have shown that deep water in wetlands is an effective barrier to terrestrial nest predators (Sanchez-Lafuente, Alcántra & Romero, 1998; Hoover, 2006). In my study I was able to confirm that daily survival rate of Bittern broods was greater, the deeper the water. This suggests that in this population the main predators were probably mammals, which unlike raptors, may have limited access to nests in reedbeds growing in deep water. Similar relationships have been found for other Bittern populations (Adamo, Puglisi & Baldaccini, 2004; Gilbert et al., 2005) and for other aquatic birds (Sanchez-Lafuente, Alcántra & Romero, 1998). This, however, is not a rule for all reedbed species. Jedlikowski, Brzeziński & Chibowski (2015) demonstrated that water depth did not significantly affect breeding success in the Water Rail *Rallus aquaticus* and Little Crake *Porzana parva*, since the main predator of their nests was a raptor, the Marsh Harrier *Circus aeruginosus*.

### Vegetation structure

Building the nest in tall, dense vegetation can be a successful strategy for alleviating predation pressure on broods (Martin, 1993; Lima, 2009): this has been demonstrated in many bird species, both shrub-nesting passerines (Goławski & Mitrus, 2008) and aquatic birds inhabiting wetland reedbeds (Kristiansen, 1998). I have shown here that where predation levels are high (Polak & Kasprzykowski, 2010), nests well concealed in dense vegetation are more likely to survive. This analysis did not indicate, however, that vegetation height was a variable significantly improving brood success: this factor is significant for populations of birds depredated by avian raptors (Jedlikowski, Brzeziński & Chibowski, 2015).

### Fragmentation of habitat patches

Ample evidence is available to show that habitat fragmentation significantly modifies the pattern of nest predation in many aquatic bird species: nests located in smaller habitat patches are more exposed to depredation (Hoover, 2006). A mosaic of diverse habitats with an extensive ecotone may make it easier for predators to gain access to nests (Caro, 2005). However, I did not observe this to be the case in this Bittern population. All nests were in relatively small reedbeds and very probably the habitat patches situated around fishponds in eastern Poland were too small to be safe nesting sites for Bitterns (see Báldi & Batáry, 2005).

## Conclusions

This work showed that the risk of nest loss in Eurasian Bitterns was the highest at the start of the breeding season. Because the reedbed stem density at this time is still rather low, females have only a small number of potential sites to choose from where they can effectively conceal their nests from predators. Among the habitat variables analysed here, two-water depth and vegetation density—were of the greatest significance in affecting nest success. In this study area, daily survival rate in Bittern broods were lower in nests built over shallow water and in thin vegetation. The present study has shown that plans for the management of this endangered species should focus on ensuring a stable, high water level in structurally diverse reedbeds. This will limit predation pressure and improve the daily nest survival rate of Bittern. Ensuring safe nesting sites for female Bitterns is particularly important during the most crucial period of reproduction, i.e., at the beginning of the breeding season.

##  Supplemental Information

10.7717/peerj.2047/supp-1 Data S1 Raw data

## Additional Information and Declarations

### Field Study Permissions

The following information was supplied relating to field study approvals (i.e., approving body and any reference numbers): The study fulfilled the current Polish Law and was permitted by Ministry of the Environment (approval number: DOPog-4201-03-48/05/al.). The Provincial Nature Conservator in Lublin allowed for this research project by the letter (number of aproval: ŚR.IV.6631-ZW / 70/2003).

### Data Availability

The following information was supplied regarding data availability: The raw data has been supplied as Data S1.
