# Supplementary material for: Nest survival patterns in Eurasian Bittern: effect of nest age, time and habitat variables
Source: PeerJ. 2016 Jun 16;4:e2047. doi: 10.7717/peerj.2047 (PMC4918471; doi:10.7717/peerj.2047)
Supplement: Data S1 [file peerj-04-2047-s001.docx]

| code nest | k (season day) | L (last day) | m (control day) | f (fate) | year | site | water | height | denisty | distow | distdyke | proare | age |
| --- | --- | --- | --- | --- | --- | --- | --- | --- | --- | --- | --- | --- | --- |
| bs03/05 | 14 | 79 | 79 | 0 | 1 | 1 | 48 | 2,4 | 36 | 25 | 8 | 5,1 | 0 |
| bs03/19 | 39 | 67 | 67 | 0 | 1 | 2 | 37 | 2 | 0 | 32 | 9 | 3,8 | 8 |
| bs03/16 | 30 | 56 | 56 | 0 | 1 | 2 | 67 | 2,8 | 13 | 16 | 12 | 4,4 | 8 |
| bs03/20 | 39 | 50 | 50 | 0 | 1 | 2 | 24 | 2,1 | 49 | 30 | 12 | 1,2 | 26 |
| bs03/21 | 50 | 56 | 56 | 0 | 1 | 2 | 10 | 1,9 | 47 | 30 | 8 | 1,2 | 28 |
| bs03/15 | 30 | 56 | 56 | 0 | 1 | 2 | 42 | 2,4 | 27 | 20 | 50 | 1,4 | 13 |
| bs03/10 | 24 | 56 | 56 | 0 | 1 | 2 | 75 | 2,1 | 59 | 20 | 60 | 1,4 | 9 |
| bs03/14 | 30 | 67 | 67 | 0 | 1 | 2 | 35 | 2,2 | 8 | 25 | 14 | 1,4 | 6 |
| bs03/13 | 30 | 50 | 60 | 1 | 1 | 2 | 48 | 2,5 | 15 | 10 | 35 | 1,4 | 6 |
| bs03/09 | 24 | 60 | 60 | 0 | 1 | 2 | 60 | 2,5 | 10 | 10 | 35 | 1,4 | 2 |
| bs03/04 | 10 | 10 | 17 | 1 | 1 | 2 | 47 | 3 | 30 | 19 | 15 | 4,1 | 0 |
| bs03/18 | 34 | 76 | 76 | 0 | 1 | 3 | 78 | 2 | 39 | 9 | 14 | 19 | 15 |
| bs03/11 | 27 | 27 | 64 | 1 | 1 | 3 | 44 | 2 | 31 | 25 | 30 | 4 | 5 |
| bs03/07 | 27 | 64 | 64 | 0 | 1 | 3 | 56 | 2 | 22 | 17 | 24 | 0,1 | 0 |
| bs03/12 | 27 | 54 | 54 | 0 | 1 | 3 | 36 | 2 | 50 | 16 | 23 | 0,1 | 0 |
| bs03/01 | 6 | 42 | 42 | 0 | 1 | 4 | 27 | 2,9 | 43 | 70 | 100 | 1 | 0 |
| bs03/17 | 31 | 53 | 53 | 0 | 1 | 4 | 53 | 3 | 0 | 25 | 20 | 0,3 | 15 |
| bs04/01 | 2 | 2 | 11 | 1 | 2 | 2 | 41 | 3,08 | 16 | 15 | 15 | 1,4 | 1 |
| bs04/02 | 5 | 47 | 47 | 0 | 2 | 4 | 55 | 3,38 | 39 | 23 | 15 | 2,1 | 1 |
| bs04/03 | 5 | 47 | 47 | 0 | 2 | 4 | 60 | 2,34 | 35 | 9 | 25 | 2,1 | 9 |
| bs04/04 | 11 | 23 | 39 | 1 | 2 | 2 | 19 | 2,5 | 0 | 13 | 15 | 3,2 | 0 |
| bs04/05 | 11 | 45 | 45 | 0 | 2 | 2 | 50 | 2,38 | 45 | 11 | 23 | 1,4 | 6 |
| bs04/06 | 17 | 17 | 44 | 1 | 2 | 5 | 32 | 2 | 48 | 39 | 8 | 0,4 | 1 |
| bs04/07 | 23 | 45 | 55 | 1 | 2 | 2 | 10 | 2,14 | 38 | 28 | 25 | 1,2 | 7 |
| bs04/08 | 23 | 29 | 39 | 1 | 2 | 2 | 22 | 2,5 | 0 | 13 | 44 | 1,2 | 6 |
| bs04/09 | 23 | 39 | 45 | 1 | 2 | 2 | 22 | 2,02 | 39 | 13 | 21 | 1,6 | 1 |
| bs04/10 | 23 | 45 | 45 | 0 | 2 | 2 | 22 | 1,74 | 40 | 13 | 65 | 1,6 | 17 |
| bs04/11 | 23 | 45 | 45 | 0 | 2 | 2 | 65 | 1,8 | 68 | 6 | 45 | 1,4 | 12 |
| bs04/12 | 23 | 45 | 55 | 1 | 2 | 2 | 33 | 2 | 0 | 10 | 8 | 1,4 | 2 |
| bs04/13 | 24 | 47 | 47 | 0 | 2 | 6 | 43 | 2,525 | 38 | 9 | 15 | 2,2 | 3 |
| bs04/14 | 24 | 30 | 38 | 1 | 2 | 4 | 50 | 2,64 | 62 | 20 | 21 | 2,1 | 8 |
| bs04/15 | 23 | 45 | 45 | 0 | 2 | 2 | 56 | 2 | 0 | 13 | 45 | 1,4 | 19 |
| bs04/16 | 29 | 29 | 39 | 1 | 2 | 2 | 25 | 2,2 | 9 | 24 | 44 | 1,2 | 7 |
| bs04/17 | 31 | 51 | 51 | 0 | 2 | 7 | 66 | 2 | 0 | 15 | 42 | 2,7 | 28 |
| bs04/18 | 31 | 31 | 51 | 1 | 2 | 7 | 69 | 2 | 0 | 10 | 25 | 2,7 | 19 |
| bs04/19 | 31 | 51 | 51 | 0 | 2 | 8 | 34 | 2,46 | 60 | 15 | 16 | 0,5 | 28 |
| bs04/20 | 26 | 61 | 61 | 0 | 2 | 3 | 32 | 2,16 | 35 | 4 | 10 | 14 | 14 |
| bs04/21 | 11 | 55 | 55 | 0 | 2 | 2 | 12 | 2,5 | 0 | 8 | 6 | 1,2 | 2 |
| bs04/22 | 39 | 39 | 46 | 1 | 2 | 2 | 13 | 2,26 | 25 | 22 | 15 | 1,2 | 5 |
| bs04/23 | 39 | 55 | 55 | 0 | 2 | 2 | 59 | 2 | 38 | 18 | 38 | 1,4 | 33 |
| bs04/25 | 44 | 57 | 57 | 0 | 2 | 3 | 49 | 3,22 | 42 | 4 | 10 | 0,1 | 35 |
| bs04/26 | 53 | 53 | 61 | 1 | 2 | 3 | 23 | 2,26 | 36 | 10 | 12 | 2 | 20 |
| bs05/01 | 1 | 1 | 9 | 1 | 3 | 2 | 42 | 2,04 | 6 | 9 | 10 | 3,5 | 0 |
| bs05/02 | 11 | 11 | 35 | 1 | 3 | 6 | 30 | 2,02 | 31 | 25 | 17 | 2,2 | 5 |
| bs05/03 | 15 | 53 | 53 | 0 | 3 | 2 | 66 | 2 | 0 | 8 | 18 | 1,3 | 10 |
| bs05/04 | 15 | 15 | 23 | 1 | 3 | 2 | 31 | 2,02 | 13 | 12 | 7 | 3,5 | 3 |
| bs05/05 | 15 | 15 | 22 | 1 | 3 | 2 | 40 | 2,5 | 27 | 15 | 20 | 3,4 | 5 |
| bs05/06 | 18 | 57 | 57 | 0 | 3 | 3 | 50 | 2,4 | 37 | 6 | 12 | 0,1 | 11 |
| bs05/07 | 18 | 18 | 30 | 1 | 3 | 5 | 33 | 2,36 | 40 | 35 | 12 | 0,4 | 4 |
| bs05/08 | 26 | 49 | 49 | 0 | 3 | 8 | 59 | 3 | 52 | 30 | 15 | 0,5 | 16 |
| bs05/09 | 26 | 37 | 37 | 0 | 3 | 7 | 41 | 2,5 | 0 | 15 | 15 | 2,7 | 18 |
| bs05/10 | 29 | 60 | 60 | 0 | 3 | 9 | 34 | 1,98 | 17 | 4 | 25 | 8,6 | 12 |
| bs05/11 | 30 | 37 | 44 | 1 | 3 | 5 | 44 | 2 | 41 | 45 | 7 | 0,4 | 3 |
| bs05/12 | 36 | 60 | 60 | 0 | 3 | 2 | 64 | 2,5 | 24 | 8 | 30 | 1,2 | 20 |
| bs05/13 | 39 | 77 | 77 | 0 | 3 | 4 | 97 | 2 | 0 | 20 | 10 | 0,3 | 22 |
| bs05/14 | 39 | 77 | 77 | 0 | 3 | 4 | 94 | 2 | 0 | 15 | 15 | 0,3 | 34 |
| bs05/15 | 39 | 77 | 77 | 0 | 3 | 4 | 82 | 2 | 0 | 20 | 8 | 0,3 | 23 |
| bs05/16 | 44 | 57 | 57 | 0 | 3 | 5 | 36 | 3 | 48 | 45 | 7 | 0,4 | 31 |
| bs06/01 | 9 | 22 | 34 | 1 | 4 | 4 | 47 | 2,46 | 49 | 34 | 12 | 1 | 2 |
| bs06/02 | 9 | 34 | 44 | 1 | 4 | 4 | 64 | 2,06 | 31 | 53 | 93 | 1 | 3 |
| bs06/03 | 15 | 57 | 57 | 0 | 4 | 5 | 38 | 2,2 | 80 | 30 | 14 | 0,4 | 2 |
| bs06/04 | 15 | 57 | 57 | 0 | 4 | 5 | 35 | 2,5 | 72 | 40 | 10 | 0,4 | 3 |
| bs06/05 | 15 | 15 | 32 | 1 | 4 | 3 | 35 | 2,18 | 22 | 12 | 14 | 4 | 5 |
| bs06/06 | 15 | 15 | 32 | 1 | 4 | 3 | 39 | 3,2 | 50 | 6 | 12 | 4 | 0 |
| bs06/07 | 15 | 47 | 47 | 0 | 4 | 8 | 59 | 2,975 | 85 | 52 | 12 | 0,5 | 9 |
| bs06/08 | 15 | 52 | 52 | 0 | 4 | 7 | 61 | 2 | 0 | 30 | 35 | 2,7 | 7 |
| bs06/10 | 17 | 37 | 49 | 1 | 4 | 2 | 59 | 2,78 | 32 | 25 | 14 | 3,4 | 1 |
| bs06/11 | 17 | 28 | 34 | 1 | 4 | 2 | 28 | 1,9 | 24 | 7 | 7 | 1,9 | 5 |
| bs06/12 | 17 | 49 | 49 | 0 | 4 | 2 | 68 | 2,38 | 21 | 32 | 13 | 3,4 | 10 |
| bs06/14 | 22 | 55 | 55 | 0 | 4 | 4 | 45 | 2,56 | 39 | 37 | 9 | 2,1 | 5 |
| bs06/15 | 22 | 49 | 49 | 0 | 4 | 4 | 46 | 2,62 | 32 | 27 | 9 | 2,1 | 8 |
| bs06/16 | 22 | 49 | 49 | 0 | 4 | 10 | 30 | 2,02 | 42 | 18 | 12 | 0,2 | 13 |
| bs06/17 | 32 | 57 | 57 | 0 | 4 | 5 | 46 | 2,05 | 50 | 40 | 8 | 0,4 | 5 |
| bs06/18 | 32 | 57 | 57 | 0 | 4 | 5 | 23 | 2,3 | 73 | 26 | 24 | 0,4 | 15 |
| bs06/19 | 32 | 32 | 52 | 1 | 4 | 3 | 38 | 2,96 | 23 | 29 | 16 | 0,1 | 15 |
| bs06/21 | 32 | 47 | 47 | 0 | 4 | 7 | 62 | 2 | 0 | 50 | 45 | 2,7 | 27 |
| bs06/22 | 32 | 52 | 52 | 0 | 4 | 7 | 52 | 2 | 0 | 45 | 20 | 2,7 | 17 |
| bs07/01 | 6 | 6 | 27 | 1 | 5 | 2 | 35 | 2,42 | 43 | 21 | 17 | 1,2 | 2 |
| bs07/03 | 8 | 32 | 32 | 0 | 5 | 4 | 46 | 2,6 | 67 | 70 | 100 | 1 | 13 |
| bs07/04 | 8 | 42 | 42 | 0 | 5 | 4 | 37 | 3,02 | 35 | 30 | 8 | 2,1 | 13 |
| bs07/05 | 14 | 49 | 49 | 0 | 5 | 5 | 26 | 3,1 | 69 | 28 | 8 | 0,4 | 3 |
| bs07/06 | 14 | 49 | 49 | 0 | 5 | 5 | 15 | 3,2 | 59 | 27 | 15 | 0,4 | 10 |
| bs07/07 | 14 | 33 | 49 | 1 | 5 | 3 | 22 | 1,88 | 30 | 29 | 16 | 0,1 | 2 |
| bs07/08 | 21 | 48 | 48 | 0 | 5 | 10 | 35 | 2,4 | 51 | 48 | 15 | 0,2 | 9 |
| bs07/09 | 21 | 48 | 48 | 0 | 5 | 10 | 33 | 2,54 | 50 | 46 | 18 | 0,2 | 16 |
| bs07/11 | 27 | 59 | 59 | 0 | 5 | 2 | 59 | 2,86 | 40 | 23 | 6 | 1,3 | 3 |
| bs07/12 | 34 | 34 | 62 | 1 | 5 | 9 | 64 | 3 | 46 | 7 | 20 | 8,6 | 5 |
| bs07/13 | 36 | 59 | 59 | 0 | 5 | 2 | 61 | 2,46 | 35 | 10 | 12 | 4,4 | 12 |
| bs07/14 | 36 | 59 | 59 | 0 | 5 | 2 | 67 | 2,5 | 33 | 15 | 15 | 4,4 | 11 |
| bs08/01 | 11 | 37 | 37 | 0 | 6 | 10 | 32 | 1,9 | 44 | 30 | 20 | 0,2 | 14 |
| bs08/02 | 19 | 47 | 47 | 0 | 6 | 2 | 42 | 2,32 | 29 | 15 | 20 | 1,5 | 16 |
| bs08/03 | 24 | 37 | 37 | 0 | 6 | 4 | 68 | 2,5 | 16 | 12 | 16 | 2,1 | 30 |
| bs08/04 | 24 | 47 | 47 | 0 | 6 | 4 | 52 | 3 | 42 | 22 | 17 | 2,1 | 15 |
| bs08/05 | 30 | 31 | 31 | 0 | 6 | 3 | 35 | 2,18 | 22 | 15 | 20 | 0,1 | 30 |
